# Exploring the effect of a sweltering environment on the risk of death from cardiovascular diseases

**DOI:** 10.3389/fneur.2024.1481384

**Published:** 2024-12-20

**Authors:** Zhaocong Chen, Wangchao Li, Zhengjie Zhu, Xueliang Miao, Shuai Jiang, Caiming Li

**Affiliations:** ^1^The Second Clinical Medical College of Guangdong Medical University, Dongguan, Guangdong, China; ^2^Huizhou Meteorological Bureau, Huizhou, Guangdong, China; ^3^Huizhou City Emergency Warning Information Release Center, Huizhou, China; ^4^Department of Neurology, Huizhou First Hospital, Huizhou, China

**Keywords:** sweltering conditions, temperature-humidity index, cardiovascular diseases, stroke, cumulative lag

## Abstract

**Background:**

A substantial body of research has demonstrated a notable impact of hot temperatures on mortality from cardiovascular diseases (CVDs). However, a paucity of studies has addressed the influence of sweltering conditions on CVD mortality.

**Objective:**

To investigate the effect of sweltering conditions on mortality from CVD among permanent residents of Huizhou City, using the temperature-humidity index (THI) as an indicator.

**Methods:**

This study employs descriptive statistics, distributed lag non-linear model (DLNM) and general algebraic modeling system (GAMs) with the THI as an indicator in order to examine the impact of sweltering conditions on the mortality of CVD among permanent residents of Huizhou City.

**Results:**

Sweltering conditions increase the risk of death from CVDs and have a cumulative lag effect. The greater the THI, the more pronounced the increase in mortality, and after a certain range, the mortality rate from CVDs increases significantly, and the effect is gender-specific. The lag effect generally peaks in 2–3 days, and the lag effect of stroke mortality is longer and deeper than that of coronary heart disease (CAD) mortality.

**Conclusion:**

Sweltering increased the mortality of cardiovascular diseases in Huizhou city, so we should pay attention to public health intervention strategies under sweltering.

## 1 Introduction

A significant number of prevalent human diseases are linked to climate fluctuations, and warming trends in recent decades have led to increased morbidity and mortality from diseases such as CVDs in many parts of the world (1–5). A meta-analysis of studies has demonstrated that CVD-related mortality increases with increasing ambient temperature, and that the risk of death from stroke increases by 3.8 per cent and the risk of death from CAD by 2.8 per cent for every 1°C rise in ambient temperature, and has demonstrated that the risk of CVD varies geographically and is affected by a number of underlying climatic conditions (6).

To date, a large amount of literature has confirmed a strong correlation between high temperatures and increased mortality from CVD (7–10). However, most of these studies only focused on the impact of temperature as an influencing factor on the human body, while humidity was controlled as a confounding factor (11). With global warming, the earth’s climate is becoming warmer and wetter, and focusing only on temperature or humidity can no longer better quantify the impact of climate change on CVD. The complexity of the impact of climate on disease makes it challenging to study the relationship of a single factor in isolation, and because there is often a joint effect between the factors, a single-factor analysis cannot accurately reflect the real climate situation.

Physiologically, it has been confirmed that high humidity at high temperatures can prevent the cooling effect of the cooling system. In a hot and humid environment, high humidity reduces the body’s own cooling ability (12–15), which can lead to an increase in the body’s core temperature and in turn put a strain on the cardiovascular system (16). On this basis, some scholars have begun to suggest that there may be a combined effect between temperature and humidity, and that this effect may exacerbate the damage to the cardiovascular system caused by high temperatures, leading to an increased risk of death (17, 18). Of course, some scholars believe that high humidity in hot conditions may be a protective factor (19, 20). To date, these conclusions are inconsistent, highlighting the need for a systematic investigation of the joint effects of humidity and temperature on CVD mortality under sweltering conditions.

The effects of damp heat in various regions are likely to vary due to weather conditions, air pollution, socioeconomic status and demographic characteristics. Our study aims to use a dataset from Huizhou City, Guangdong Province, to investigate the combined effects of relative humidity and high temperature on CVD mortality, and to generalize the results to subtropical monsoon humid climate zones, especially those that experience hot and humid weather year-round. This will enable us to protect people at risk of cardiovascular disease and reduce their exposure to risk in advance of hot and humid weather, so as to prevent CVD mortality.

## 2 Materials and methods

### 2.1 Research environment

This study was conducted in Huizhou City, Guangdong Province, which is located between 22°24′ and 23°57′ north latitude and 113°51′ and 115°28′ east longitude, in the south of China, with a population of about 6,042,900 people. The region falls within a typical subtropical monsoon humid climate zone, with a mean annual precipitation of 1,770 mm mainly from May to September and a mean annual temperature of 22°C, with the highest temperature in summer often reaching over 30°C.

### 2.2 Data collection

The mortality data of permanent residents in Huizhou from 2015 to 2021 were retrieved from the death information registration and management department of Huizhou City. The mortality data encompassed fundamental individual information, such as gender, age, time of death, and cause of death. In accordance with the International Classification of Diseases, 10th revision (ICD-10, coding: I00-I99), the mortality data of CVDs were extracted, and on this basis, CAD and stroke were further screened out. All the above data is divided and analyzed in units of days.

The meteorological data of the same period are from the Huizhou Meteorological Information Centre, including daily mean temperature (°C), daily maximum temperature (°C), daily mean wind speed (m/s), daily mean relative humidity (%), etc.

### 2.3 Statistical analysis

#### 2.3.1 Temperature-humidity index

Temperature-humidity index (THI) has been employed extensively in China since the advent of the 21st century, primarily as an indicator of human comfort. While research has been conducted on the impact of temperature and humidity on CVDs, there have been few attempts to assess these effects using a recognized comprehensive index. As a well-established and widely utilized index, THI is particularly suited to this study. Its calculation formula is (21):


(1) $$THI=\left(1.8T+32\right)-0.55\times \left(1-RH\right)\times \left(1.8T-26\right)$$

In equation 1: THI-temperature-humidity index; T-daily mean temperature; RH-relative humidity. When THI ≥ 75, it is defined as sweltering. Calculations and statistics are based on the above formula and the standard (Supplementary Table S1).

#### 2.3.2 Data selection

The annual distribution of THI was obtained by analyzing the death number of CVDs and meteorological element data in Huizhou from 2015 to 2021 (Supplementary Figure S1), and it was found that THI ≥ 75 mainly appeared from May to September each year. Therefore, the scope of the study was narrowed to include May to September as the focused analysis period.

#### 2.3.3 Statistical methods

##### 2.3.3.1 Descriptive statistics

This study describes and analyses data on deaths from CVDs and meteorological data for residents of Huizhou City from 2015 to 2021, and calculates the mean, variance, minimum, quartile, maximum and other values of each indicator.

##### 2.3.3.2 Time series analysis

The general algebraic modeling system (GAMs) is suitable for analyzing complex nonlinear relationships between dependent variables and several explanatory variables. It is widely used in epidemiology and environmental health. The explanatory variables can be fitted using various smoothing functions to represent the degree of influence of each explanatory variable on the dependent variable. Since the effect of changes in THI on the risk of CVDs mortality is not limited to the observed time period and may also have a certain lag, the distributed hysteresis nonlinear model (DLNM) proposed by Gasparrini is introduced to model the relationship between exposure events and a series of future outcomes (22). Therefore, this study used a Poisson distribution GAM combined with DLNM to assess the association between THI and the risk of CVDs mortality in residents. Before establishing the model association, in order to avoid the collinearity of the factors in the model, the Separman correlation coefficient between the meteorological factors was tested (23). If the correlation between the two factors is strong (|*r*| > 0.8) (24), it indicates that the two variables are highly collinear and should not be included in the same model.

According to the results of the Spearman correlation analysis, the control variables in this study were set to wind speed, long-term time trend, day of the week effect, and holiday effect. The GAMs formula is as follows:


(2) $$\begin{array}{l} \log\left[E\left(Y_{t}\right)\right]=\beta TI_{t-\mathrm{lag}}+s\left(time_{t},df=7*7\right)\\ \;+s\left(WS_{t},df=3\right)+DOW+\mathrm{Holiday}+\alpha \end{array}$$

In equation 2: Y-the number of CVDs deaths on day t; E(Y_t_)-the expected value of the number of CVDs deaths on day t; lag–lag time; s-cubic spline function; df-degree of freedom parameter; *α*- intercept; TI_t-lag_-THI lagged by t days; $WS_{t}$ -mean wind speed on day t; time–time variable, with 7*year selected as the degree of freedom to control for long-term temporal trends; DOW-day of the week; Holiday–holiday as a confounding factor, added as a dummy variable.

The regression coefficientβand standard deviation SE were estimated in accordance with equation 2, and the relative risk (RR) and its 95% confidence interval (95% CI) were calculated (25). Please refer to equations 3, 4 for further details:


(3) $$RR=\exp\left(10\times \beta \right)$$

(4) $$RR\%\left(95\%CI\right)=\left[\exp\left(10\times \left(\beta \pm 1.95\times SE\right)\right)\right]$$

Based on the above effects, the DLNM was constructed to predict the RR of CVDs deaths in residents under different THI values. First, a cross-base matrix was generated for the primary research factor THI, and the additional lag time dimension of the exposure-response relationship, that is, the combination of the two functions of prediction and lag effect, was combined into a two-dimensional matrix. The lag dimension was set to 7 days (26, 27), and the model framework was as follows:


$$\begin{array}{l} \log\left[E\left(Y_{t}\right)\right]=cb\left(TI_{t},\mathrm{lag}\right)+s\left(time_{t},df=7*7\right)\\ \;+s\left(WS_{t},df=3\right)+DOW+\mathrm{Holiday}+\alpha \end{array}$$

In the above formula: cb-cross-base matrix, where the three internal nodes are the 10th, 75th, and 90th percentiles of the temperature distribution; and polynomial functions are used to construct its lagged effects, with the maximum number of lagged days set to 7 d.

#### 2.3.4 Sensitivity analyses

To test the sensitivity of the model and the effect of THI in this study, the following Sensitivity analyses were performed to demonstrate the robustness of our model formulation: 1. Changing the time degrees of freedom 7*7; 2. Including CVD, stroke and CAD death data into the model for testing separately. The results calculated under different degrees of freedom were subjected to a significance t-test with *α* = 0.05 against the data from the main model. *p* < 0.05 indicates a statistical difference.

All statistical analyses in this study were performed using R4.4.1 software, and the mgcv., dlnm, and ggplot2 packages were used to assess the impact of sweltering on the number of deaths from CVD and the two core disease types in different genders, as well as the cumulative lag effect and data visualization. Statistical tests were two-sided probability tests, with a test standard of *α* = 0.05. All results are expressed as RR and 95% CI, and a *p* value of <0.05 was considered statistically significant.

## 3 Results

### 3.1 Statistical analysis of CVD deaths and meteorological elements in Huizhou City from May to September

From May to September 2015–2021, 19,525 people died from cardiovascular and cerebrovascular diseases in Huizhou (male: 10,144; female: 9,381). Total deaths from stroke: 7,521 (male 3,893; female 3,628), total deaths from coronary heart disease: 7,769 (male 4,155; female 3,614). The specific statistical characteristics of the number of CVD deaths and meteorological factors in Huizhou City are shown in Table 1.

**Table 1:** Descriptive statistics of daily CVDs mortality, THI, mean temperature, mean wind speed and relative humidity during 2015–2021 in Huizhou, China.

	Variables	Mean	SD	Min	1	10	25	50	75	90	99	Max
CVDs mortality	Total	8.2	5.1	5.0	8.0	2.0	5.0	8.0	1.0	5.0	1.0	36.0
	Men	9.5	3.3	1.0	2.7	5.0	7.0	9.0	2.0	4.0	7.3	25.0
	Women	8.8	3.3	0.0	3.0	5.0	6.0	8.0	11.0	13.0	17.0	20.0
	0–64 years	3.0	1.9	0.0	0.0	1.0	2.0	3.0	4.0	5.0	9.0	11.0
	≥65 years	15.3	4.7	4.0	6.0	9.0	12.0	15.0	18.0	21.0	28.0	32.0
Disease classification	Coronary artery disease	7.3	2.9	0.0	1.0	4.0	5.0	7.0	9.0	11.0	15.0	18.0
	Stroke	7.0	3.1	0.0	0.7	3.0	5.0	7.0	9.0	11.0	16.0	18.0
Meteorological elements	THI	79.6	2.7	65.3	71.1	76.1	78.1	80.2	81.5	82.5	83.7	84.9
	MT (°C)	27.9	2.0	18.5	22.3	25.3	26.6	28.1	29.5	30.2	31.4	32.1
	MWS (m/s)	2.0	0.7	0.4	0.9	1.3	1.6	1.9	2.4	2.9	3.8	9.3
	RH (%)	81.2	8.8	43.0	61.0	70.9	75.0	80.0	88.0	94.0	98.3	99.0

CVDs, Cardiovascular diseases; MT, mean temperature; MWS, mean wind speed; RH, relative humidity.

According to our preliminary statistics on the number of sweltering conditions days and the corresponding number of deaths, it was concluded that the number of sweltering conditions days during 2015–2021 was 1,012 d, with an annual mean of 144.6 d. The mean daily number of CVD deaths during the sweltering conditions period was 18.5, of which 9.5 were males and 8.8 were females. This is higher than the mean daily number of deaths during non-sweltering conditions.

### 3.2 Using GAM to analyze the effects and lagged effects of sweltering on CVD and mortality of two core diseases in different genders

The cumulative lag effect of Sweltering on CVDs mortality from May to September, 2015 to 2021 is shown in Table 2. In terms of the overall effect, the cumulative lag effect of Sweltering on CVDs mortality is more significant, and the effect on women is more significant than that on men.

**Table 2:** Effects of sweltering on cardiovascular diseases mortality from 2015 to 2021, stratified by sex.

Effect	Lag of day	RR (95%CI)		
		Total	Men	Women
Sweltering	0	1.030 (1.022–1.039)***	1.027 (1.015–1.039)***	1.034 (1.021–1.046)***
	1	1.038 (1.028–1.047)***	1.034 (1.021–1.047)***	1.042 (1.029–1.055)***
	2	1.039 (1.028–1.049)***	1.035 (1.020–1.049)***	1.043 (1.029–1.058)***
	3	1.036 (1.025–1.047)***	1.033 (1.017–1.049)***	1.040 (1.024–1.055)***
	4	1.031 (1.019–1.043)***	1.028 (1.011–1.045)***	1.034 (1.018–1.051)***
	5	1.026 (1.013–1.039)***	1.022 (1.004–1.040)*	1.031 (1.013–1.049)***
	6	1.022 (1.008–1.036)**	1.017 (0.998–1.036)	1.028 (1.009–1.047)**
	7	1.018 (1.003–1.033)*	1.014 (0.994–1.035)	1.024 (1.004–1.045)*

*p < 0.05; **p < 0.01; ***p < 0.001.

Second, in a separate analysis of the data on deaths from coronary heart disease (Tables 3, 4), the risk of death from coronary heart disease increased with the cumulative sweltering effect for both men and women. The effect of perceived sweltering on coronary heart disease in men peaked at lag 1, with a 2.8% increase in mortality (RR, 1.028; 95% CI [1.009–1.048]), while in women the peak was reached on the cumulative lag day 2, with an increase in mortality of 3.5% (RR, 1.035; 95% CI [1.015–1.054]).

**Table 3:** Effects of sweltering on coronary artery disease mortality from 2015 to 2021, stratified by sex.

Effect	Lag of day	RR (95%CI)		
		Total	Men	Women
Sweltering	0	1.029 (1.017–1.042)***	1.025 (1.007–1.043)**	1.027 (1.010–1.044)***
	1	1.036 (1.022–1.050)***	1.028 (1.009–1.048)**	1.033 (1.015–1.052)***
	2	1.033 (1.018–1.048)***	1.020 (1.000–1.041)	1.035 (1.015–1.054)***
	3	1.029 (1.012–1.045)***	1.014 (0.992–1.037)	1.032 (1.011–1.053)**
	4	1.024 (1.007–1.042)**	1.009 (0.986–1.033)	1.028 (1.007–1.050)**
	5	1.020 (1.002–1.039)*	1.004 (0.979–1.029)	1.026 (1.004–1.048)*
	6	1.017 (0.998–1.037)	1.000 (0.974–1.026)	1.023 (1.000–1.046)
	7	1.015 (0.995–1.036)	1.000 (0.973–1.028)	1.018 (0.995–1.042)

*p < 0.05; **p < 0.01; ***p < 0.001.

**Table 4:** Effects of sweltering on stroke mortality from 2015 to 2021, stratified by sex.

Effect	Lag of day	RR (95%CI)		
		Total	Men	Women
Sweltering	0	1.031 (1.018–1.045)***	1.027 (1.008–1.046)**	1.036 (1.016–1.055)***
	1	1.041 (1.026–1.056)***	1.038 (1.017–1.060)***	1.045 (1.024–1.067)***
	2	1.046 (1.030–1.063)***	1.049 (1.026–1.073)***	1.046 (1.023–1.069)***
	3	1.046 (1.029–1.064)***	1.054 (1.029–1.079)***	1.042 (1.017–1.067)***
	4	1.044 (1.025–1.062)***	1.051 (1.024–1.078)***	1.039 (1.013–1.066)**
	5	1.039 (1.020–1.059)***	1.047 (1.018–1.076)**	1.035 (1.007–1.063)*
	6	1.033 (1.013–1.054)**	1.038 (1.008–1.069)*	1.030 (1.000–1.060)*
	7	1.027 (1.006–1.049)*	1.030 (0.999–1.063)	1.026 (0.994–1.058)

*p < 0.05; **p < 0.01; ***p < 0.001.

The effect of perceived sweltering on the risk of death from stroke also had a cumulative effect. The effect of perceived sweltering on stroke in the total population peaked on day 2 of the lag, with an increase in mortality rate of 4.6%. Among men, the effect of perceived sweltering on stroke peaked on day 3 of the lag, with an increase in mortality rate of 5.4% (RR, 1.054; 95% CI [1.029–1.079]); the effect of feeling sweltering on stroke in women peaked on the second day of the lag, with an increase in mortality of 4.6% (RR, 1.046; 95% CI [1.023–1.069]). This may indicate that the effect of sweltering on stroke in men may be more serious and long-lasting. At the same time, by comparing Tables 3, 4, it can be found that the lagged effect of stroke mortality is longer and more severe than that of coronary heart disease mortality in the general population.

### 3.3 Predicting the impact of different perceived sweltering indices on CVD mortality and their cumulative lag effects

**Figure 1:**
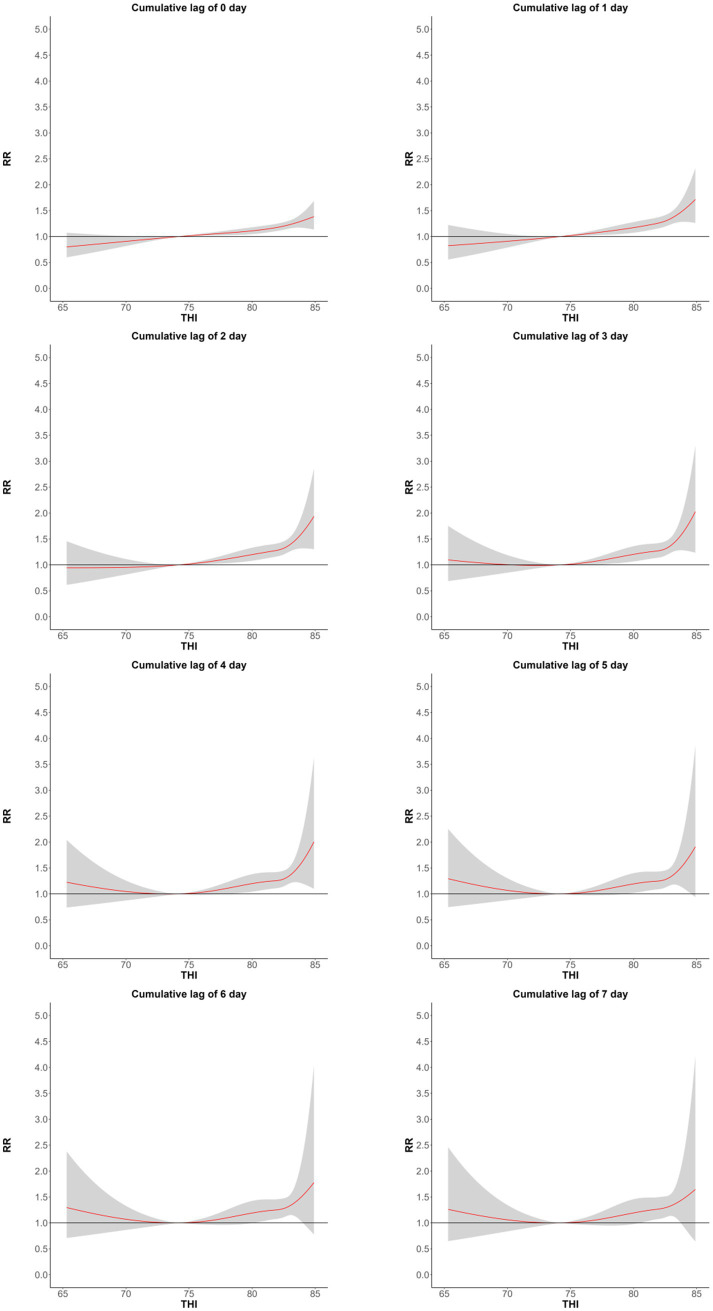
The cumulative lag effects of the relationship between the THI index and the number of CVDs deaths in Huizhou, China from May to September in the years 2015–2021, with a lag of 0–7 days.

There existed an evident nonlinear connection between THI and RR (Figure 1). When THI equaled 74.2 (corresponding to the minimum number of deaths), the average RR was the lowest, and subsequently, RR rose along with THI. Once THI exceeded a specific range, RR increased rapidly as THI increased. Furthermore, the impact of sweltering (THI > 75) on the human body possesses a cumulative lag effect. With the accumulation of the sweltering effect, the risk of CVDs death also accumulates, and the death risk reaches the peak on the 2nd lag day.

### 3.4 Sensitivity analyses

As shown in Supplementary Table S2, after adjusting for the degree of freedom of the long-term time trend, the impact of THI on CVD and mortality rates of the two core diseases did not change significantly, and the result of the t-test was *p* > 0.05, indicating that there was no significant difference between the changes in the data and that the model used in this study was reliable.

## 4 Discussion

This study first used the GAM to analyze the impact and lag effect of sweltering on CVD and mortality rates of two core diseases in different gender groups. It was found that the risk of CVD mortality in the total population increased by 3.0% under sweltering; and the RR showed a trend of first increasing and then decreasing with the increase in the number of lag days. In terms of cardiovascular disease, women showed more sensitivity; in terms of cerebrovascular disease, men showed more sensitivity. Then, DLNM was further used to predict the impact of different sweltering indices on the population of CVD mortality and their lag effects. These findings highlight the need to strengthen the prevention and treatment of cardiovascular and cerebrovascular diseases when sweltering weather occurs. At present, many literatures have confirmed various mechanisms to explain the increase in body temperature caused by the imbalance of the body caused by hot weather, which in turn leads to an increased risk of death from cardiovascular and cerebrovascular diseases (6). In a hot and humid environment, the body’s heat dissipation is limited (11–13), which is more likely to lead to an increase in body temperature after the body temperature is out of balance. The increase in body temperature after the body temperature is out of balance will ultimately lead to vascular damage and trigger the coagulation/fibrinolysis pathway (28, 29). These physiological changes may lead to microvascular thrombosis or excessive bleeding, resulting in an increased risk of ischemic stroke and heart disease. In addition, high temperatures can lead to the destabilization of blood vessel plaques (30) and accelerate the progression of atherosclerosis, increasing the risk of acute coronary syndrome (31–34).

Previous studies have also observed a strong correlation between high temperatures and mortality from CVDs. A meta-analysis of 266 studies showed that for every 1°C above the reference temperature, the risk of mortality from CVDs increased by 2.1%, and the risk of CAD increased by 2.8% (19), a retrospective study by Luo Q found that high temperatures increased CVD mortality by 3% (10). Increased body temperature increases the risk of cardiovascular dysfunction (35); Reduces coronary blood flow (36), High body temperature can also cause heart muscle damage shortly after exposure to heat (37). However, for women, the risk of death from coronary heart disease increases significantly with the cumulative sweltering effect. The research results of Zhao et al. also show that in extreme heat, coronary heart disease is more sensitive in women than in men (38).

In addition, this study also independently proves the strong correlation between sweltering and stroke and CAD mortality. Similar results have also been observed in recent studies. In the study by Luo et al., every increase of 1°C above the reference temperature increased the cerebrovascular mortality rate by 2% (10), in this meta-analysis, it was observed that for every 1°C increase in temperature above the reference temperature, the risk of stroke increased by 3.8% (19), high temperatures have been shown to be a risk factor for ischaemic stroke (IS) (39), the higher the temperature of the brain, the greater the extent of the cerebral infarction (40). A retrospective article on animal models of high-temperature-induced cerebral ischaemia explains the specific molecular mechanisms of high-temperature-induced cerebral ischaemia: for example (1) more extensive disruption of the blood–brain barrier (23, 24); (2) The number of potentially damaging ischaemic depolarizations in the ischaemic penumbra increases (41–43). Our results also show that the impact of sweltering on stroke is more significant than CAD, which is consistent with the research results of Liu et al. (19). However, there are differences with the research results of Luo et al. (10, 44). The blood–brain barrier is very sensitive to temperature changes in the event of cerebral ischaemia, and high temperatures can lead to widespread damage to the blood–brain barrier (45, 46), and after the blood–brain barrier is damaged, the accumulation of water in the brain and changes in ion homeostasis can aggravate heat injury (47). In addition, the loss of the blood–brain barrier leads to an imbalance in the immune system of the central nervous system, and the associated inflammatory response can further aggravate the deterioration of the stroke (48).

There is a cumulative lag effect on the mortality rate of CVDs due to sweltering conditions. The effect of sweltering conditions on CVDs in women is greater than that in men. Mesdaghinia et al. showed that the short-term effect of heat exposure on the risk of CVDs in men was 1.1% (RR, 1.011; 95% CI [1.009–1.013]), and 1.4% (RR, 1.014; 95% CI [1.011–1.017]) for women 38. The cumulative lag effect of sweltering is more pronounced in women than in men for CVDs.

Finally, the images in this study are different from the exposure-response curve images in the literature related to the relationship between high temperatures alone and CVDs (49). The images in this study are divided into two sections. In the first half of the images, as the THI increases, the risk of CVDs death increases slowly. Considering that when the ambient temperature is not too high, the body can still dissipate heat through heat radiation, even if the high humidity environment hinders the discharge of sweat, the body is not prone to body temperature imbalance. In the second half of the graph, as the THI increases, the body finds it difficult to cool down through heat radiation in a high-temperature environment. The body’s temperature is mostly cooled down by sweating, but in a high-humidity environment, the excretion of sweat is significantly hindered, which greatly reduces the body’s cooling efficiency in the same high-humidity environment, increasing the risk of elevated body temperature and, in turn, increasing the risk of CVDs (50), This result is consistent with the effect of wet bulb temperature on mortality. As the wet bulb temperature increases, the risk of human death also increases. When the wet bulb temperature reaches 35°C, the human cooling mechanism fails (11–13).

As global temperature continues to rise, the impact of humidity and heat on CVD becomes stronger and stronger (9). How to effectively prevent and control CVD, especially under what specific weather conditions need to be strengthened, is an urgent issue to be addressed nowadays. Our study site is located in Huizhou City, Guangdong Province, China, which has a humid subtropical monsoon climate. Studying the effect of sweltering conditions on CVD mortality in this location is an important reference for research in subtropical monsoon humid climate zones.

There are still several constraints in our investigation. Initially, our primary emphasis was on examining the correlation between sweltering conditions and cardiovascular disease mortality specifically in Huizhou City. We did not include individuals residing in different climatic regions. Furthermore, in our investigation, we employed the ambient temperature at the outdoor detection point as a substitute for personal exposure. However, this simplified approach may result in inaccuracies in measuring exposure. It is important to acknowledge and address these limitations in future studies in order to better elucidate the correlation between meteorological conditions and CVDs.

## 5 Conclusion

Sweltering conditions can increase the risk of death from CVD, and the greater the THI, the more pronounced the increase in mortality, and beyond a certain range, the mortality rate increases significantly. There was also a gender difference in this effect, with the effect being more significant in women than in men. In addition, there is a cumulative lag effect of sweltering on CVD mortality, which generally peaks after 1–3 days. In addition, the lag effect is longer and deeper for stroke deaths than for CAD deaths. Studying the effects of sweltering on CVDs has important public health and clinical implications for the prevention of CVD deaths.

## Data Availability

The original contributions presented in the study are included in the article/Supplementary material, further inquiries can be directed to the corresponding author.
