# Supplementary material for: Exploring the effect of a sweltering environment on the risk of death from cardiovascular diseases
Source: Front Neurol. 2024 Dec 20;15:1481384. doi: 10.3389/fneur.2024.1481384 (PMC11697591; doi:10.3389/fneur.2024.1481384)
Supplement: Supplementary file 1 [file Table_1.DOCX]

| **Supplementary Table S1** Grade standards of THI | | |
| --- | --- | --- |
| ValueCol | Range of value | Somatosensory classification |
| 1 | <40 | Extremely cold |
| 2 | 40-45 | Chilly |
| 3 | 45-55 | Cold |
| 4 | 55-60 | Tending toward cool |
| 5 | 60-65 | Clear and cool |
| 6 | 65-70 | Warm |
| 7 | 70-75 | Tending toward heat |
| 8 | 75-80 | sweltering |
| 9 | >80 | Extremely sweltering |
| THI,temperature-humidity index | | |


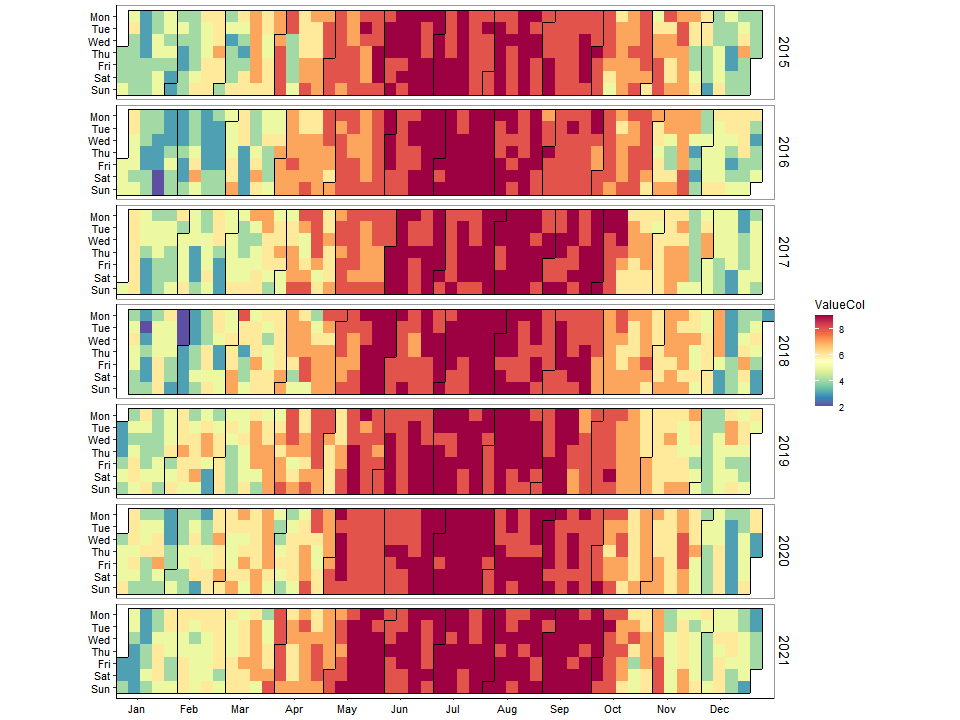


Supplementary Figure S1. Annual distribution of THI from 2015 to 2021

| **Supplementary Table S2** The impact of sweltering lag adjusted by Degree of freedom confounding factor on the number of deaths of cardiovascular diseases and two core diseases. | | | | |
| --- | --- | --- | --- | --- |
| Model parameter | RR（95%CI） | | | P-value |
|  | cardiovascular diseases | coronary artery disease | stroke |  |
| Main model | 1.030（1.022—1.039）*** | 1.029（1.017—1.042）*** | 1.031（1.018—1.045）*** |  |
| Degree of freedom=5 | 1.031（1.022—1.039）*** | 1.030（1.017—1.043）*** | 1.031（1.018—1.045）*** | 0.6781 |
| Degree of freedom=6 | 1.030（1.022—1.039）*** | 1.029（1.017—1.043）*** | 1.031（1.018—1.045）*** | 0.9649 |
| Degree of freedom=8 | 1.030（1.021—1.039）*** | 1.029（1.017—1.042）*** | 1.031（1.018—1.045）*** | 0.9421 |
| Degree of freedom=9 | 1.030（1.021—1.039）*** | 1.030（1.017—1.043）*** | 1.032（1.018—1.045）*** | 0.8125 |
| Note: Main model: Degree of freedom=7; *, P < 0.05; **, P < 0.01; ***, P < 0.001 | | | | |
